# Rapid Operations

**Published:** 1891-12

**Authors:** Frank Perrin


### RAPID OPERATIONS.[1]

BY FRANK PERRIN, D.M.D.

In discussing this subject I would first make a distinction between rapidity and hurry. By the former we understand quickness without confusion; by the latter we understand haste without collected and well-directed thought, which in careful operations must be avoided.

In what I have to say I hope you will not get the idea that it is well to rush dental operations in an undignified manner, giving the patient the idea that your aim is to get through with him as quickly as possible, and be about something else. Still nearly all will realize that the patient will appreciate quickness, and be glad to make his visit to the dentist of as short duration as safety and good work will allow.

The man of business, whose time is crowded to its limit, and who has his own affairs arranged on a time-saving basis, who daily converses over the wire with his friend in New York, or who may telegraph to his agent in Calcutta at four o'clock in the afternoon, and finds the reply on his desk on his return to his office the next morning, easily loses patience when seeing the dentist wasting time. The woman whose affairs at home demand her speedy return, or whose social circle claims her time, soon becomes nervous if she has reason to think the dentist is dilatory.

Everything in the world around us indicates that the age in which we live requires our greatest possibilities, especially when we have not only our own time but that of others in our keeping; it is our duty to use that time to its best advantage, and to use as little as possible.

Even if our patient's time is not worth a dollar to him, the question of our own still remains, and duty to self demands that we make as many dollars in the hours devoted to business as we honestly can. Moreover, what greater satisfaction can a man have to repay him for a day of close application to business than the consciousness that he has accomplished all he could reasonably expect, and by making the best use of his time he has two hours left for recreation.

The art of making haste without hurrying, or of accomplishing the greatest amount of work in a given period is a study, per-

---

[1] Read before the Harvard Odontological Society, May 28, 1891.

haps a natural accomplishment possessed by some, while to others much careful thought and application are necessary for its attainment.

The first requirement is that the operator's mind be trained to act promptly and with precision. Much time is wasted unless he is able to quickly and correctly map out each stage of the operation at hand in such a way that every step taken is progressive, and each stroke a telling one. After a few seconds thoughtful observation he must decide on the method of procedure, and the instruments best adapted for the purpose, and as far as possible place them for use.

I will venture to suggest a few points in regard to the technique of operating, knowing they must be modified to suit the special requirements of the individual.

The selection and arrangement of instruments is perhaps the first consideration, and I think the number of them should be reduced as much as possible. The young operator is pretty sure to have in his cabinet a great many forms of instruments that are not useful to him. He should by careful study adopt the use of those he finds will best aid him in his work, and remove from the drawers every one he finds not specially adapted to his methods. If this be not done his cabinet will become encumbered with instruments, many of which are never used, and serve only to obstruct and confuse the mind. I do not mean by this to discourage the trial of new forms; this should constantly be done, and their use adopted when found satisfactory.

Engine burrs should be kept in regular order,—a row in the rack for each form, graded according to size.

The use of an assistant can be brought to an almost unlimited state of perfection. A wide-awake boy or girl, who has been well trained, may in various ways save the dentist's time, taking care of instruments, washing them, and keeping them in their places, and passing the required ones wanted during an operation; prepare the filling materials for use, passing medicine and other preparations, which the operator should call by an affixed number, thus in many cases avoiding the unpleasant disturbance of the patient's mind by hearing a drug of fearful name asked for; prepare absorbent materials, assist in adjusting the dam, etc.

Ventilation of the operating-rooms is also desirable, which the assistant can attend to as each patient is dismissed. The last and most important point I shall mention, and upon which all else depends, is that of health. A dentist must not expect to accomplish much if by any reason his physical strength is at a low ebb. Mind and body are alike strained, and the nervous energy often severely taxed.

There is a limit beyond which it is impossible to go. The ambitious man must carefully watch his strength and regulate his practice to suit his ability to bear. A dentist may have strength enough to enable him to continue at work, but in order to do this rapidly and well he must have a certain amount of reserved power and energy.

The mental forces should be allowed relaxation at regular intervals. To accomplish this an entire change of thought is required, which may be obtained by various means. We would suggest activities which may give direct play to other faculties than those used in the practice of our profession,—music, painting, botany, or even the much-followed photography. These will vary the line of thought, and give rest to overworked nerves, besides developing faculties otherwise unused. Physical exercise should be taken daily in some form,—by gymnasium practice, bicycle or horse-back riding. And not only does the dentist himself reap the benefit of suitable recreation but his patient is also affected. Overstrained nerves produce irritability which will act directly on many individuals. In fact one's physical and nervous condition may be reflected and observed in the condition of another as quickly as the administration of an anæsthetic. Anxiety of mind is also easily reflected by the patient.

The power of a healthy, well-balanced mind over one which often has been strained and racked before coming to ask our assistance is as yet not fully realized.

To have at his command a supply of reserved vigor will enable the dentist to push to successful and rapid completion a difficult and tedious operation, which must drag if he has not received a supply of strength and inspiration within twenty-four hours.

Many points which may be used to increase rapidity I have not mentioned, but some of these simple suggestions will help if we apply them.

